# Correction: NiCo_2_O_4_ nanoparticles inlaid on sulphur and nitrogen doped and co-doped rGO sheets as efficient electrocatalysts for the oxygen evolution and methanol oxidation reactions

**DOI:** 10.1039/d1na90051j

**Published:** 2021-05-26

**Authors:** A. Rebekah, C. Viswanathan, N. Ponpandian

**Affiliations:** Department of Nanoscience and Technology, Bharathiar University Coimbatore-641046 India ponpandian@buc.edu.in +91-422-2422-387 +91-422-2428-421

## Abstract

Correction for ‘NiCo_2_O_4_ nanoparticles inlaid on sulphur and nitrogen doped and co-doped rGO sheets as efficient electrocatalysts for the oxygen evolution and methanol oxidation reactions’ by A. Rebekah *et al.*, *Nanoscale Adv.*, 2021, DOI: 10.1039/d1na00135c.

The authors regret that the name of the first author (A. Rebekah) was incorrectly given in the original article. The corrected name is given here.

The Royal Society of Chemistry apologises for these errors and any consequent inconvenience to authors and readers.

## Supplementary Material

